# Factors shaping sugar metabolism in Neotropical bats: A physiological and ecological perspective

**DOI:** 10.1371/journal.pone.0354755

**Published:** 2026-08-11

**Authors:** Andrea Bernal-Rivera, Cristian Calvache-Sánchez, Oscar E. Murillo-García

**Affiliations:** 1 Grupo de Investigación en Ecología Animal, Departamento de Biología, Universidad del Valle, Cali, Colombia; 2 Calima, Fundación para la Investigación de la Biodiversidad y Conservación en el Trópico, Cali, Colombia; 3 Corporación para la Gestión Ambiental Biodiversa, Cali, Valle del Cauca, Colombia; Universidad de Guadalajara, MEXICO

## Abstract

Understanding physiological constraints on adaptive capacity is essential for predicting species responses to environmental change. Sugar assimilation represents a critical physiological adaptation linked to dietary diversification, yet the intrinsic and ecological factors governing glucose homeostasis remain poorly understood. We used glucometry to evaluate blood glucose regulation across diverse Neotropical bat species, assessing the relative importance of diet, phylogeny, temporal dynamics, and individual traits. Fasting glucose levels were strongly associated with dietary guild and phylogenetic relatedness but independent of sex and body mass. Sugar assimilation capacity varied primarily with sugar type (monosaccharides versus disaccharides), dietary specialization, and temporal scale (diurnal and seasonal cycles), although omnivorous species exhibited high individual-level variability. Critically, syntopic congeners show hints of metabolic differentiation consistent with resource partitioning, suggesting physiological niche separation may reduce interspecific competition. Seasonal plasticity in sugar metabolism—particularly in frugivorous species tracking fruit availability—indicates flexible metabolic responses to resource fluctuations. These findings show that sugar metabolism in bats reflects evolutionary adaptation to their diets, influenced by evolutionary history and seasonal changes in resource availability. The metabolic specialization seen in dietary specialists, along with seasonal flexibility, has important implications for predicting species’ vulnerability to habitat loss and climate-driven shifts in resource timing, as specialists may be less resilient to environmental changes compared to metabolically flexible generalists.

## Introduction

Understanding how organisms adapt to environmental changes has become crucial for predicting species survival and ecosystem stability amid rapid environmental shifts. Biotic specialization is an influential force driving evolutionary diversification, enabling organisms to occupy distinct ecological niches through key innovations [1]. However, phylogenetically related taxa exhibit highly idiosyncratic responses to similar environmental pressures, making research on diversity, evolutionary adaptation, and trait plasticity essential for understanding the mechanisms underlying adaptive variation [2]. Diverse groups are key for understanding variability in ecological, anatomical, and physiological traits [3]. The extraordinary dietary diversity of bats—spanning insectivory, sanguivory, nectarivory, and frugivory—represents one of the most remarkable adaptive radiations in mammalian evolution [2,4–7]. This trophic diversification has driven profound physiological specializations, particularly in sugar metabolism, where species exhibit striking differences in glucose homeostasis and carbohydrate assimilation rates [7–9]. Understanding these metabolic adaptations is crucial for predicting how bat communities will respond to changes in resource availability driven by rapid environmental change.

Sugar assimilation is critical to understanding metabolic adaptation since sugar processing varies dramatically across bat dietary preferences [8–10]. Nectarivorous and frugivorous species have evolved enhanced sugar assimilation capabilities to exploit high-carbohydrate resources. In contrast, insectivorous species show reduced carbohydrate processing efficiency consistent with their diets rich in proteins and lipids [11–13]. The sugar assimilation process entails nutrient breakdown by specific enzymes and has been interplaying with the morphology of organs such as the pancreas and the intestine to perform specific functions, resulting in energy acquisition [9]. Thus, because maintaining glucose homeostasis involves several steps, signals, and enzymes that vary from one organism to another, fasting glucose levels and sugar assimilation reveal significant physiological differences that are key to sustaining life [14–16]. Even though our knowledge of sugar assimilation in bats has been increasing exponentially, the relative importance of evolutionary history versus ecological factors in shaping differences in sugar metabolism remains poorly understood. It limits our ability to predict species’ susceptibility in changing environments.

To understand the physiological processes underlying diet diversification, we evaluated whether sugar metabolism primarily reflects evolutionary history, intrinsic factors (such as sex and body weight), or ecological factors (including diet, food availability, activity patterns, and community composition) by examining sugar assimilation across multiple Neotropical bat species. In bats, larger-bodied species generally exhibit lower blood glucose levels [17]. Since females in some species tend to be larger than males [18], this size difference may influence their sugar assimilation response. Furthermore, physiological processes such as gestation and lactation significantly increase the nutritional demands of females during these periods [19–21], thereby affecting how they regulate and utilize glucose in their bodies. On the other hand, some bat species exhibit dietary shifts in response to changes in resource availability between dry and rainy seasons [21,22] which can alter digestive physiology and nutrient assimilation [23] Under conditions of high competition, individuals may benefit from metabolic flexibility to rapidly regulate glucose availability and sustain flight, foraging efficiency, and prolonged activity while facing fluctuations and scarcity of food. Consequently, seasonal dietary shifts and interspecific competition could influence bats’ metabolism and potentially shape patterns of plasma glucostasis. To explore this impact on metabolism, we used different sugars because disaccharide digestion requires an additional hydrolysis step before absorption compared to monosaccharide digestion. Bat species with different diets (e.g., insectivorous versus nectarivorous) show interspecific variation in enzymatic activity [8,9,14]. However, fruit- and nectar-feeding bats generally appear to possess sufficient sucrase capacity to efficiently process sucrose-rich diets. Therefore, these differences may reflect broader variation in digestive physiology, nutrient processing, and glucose assimilation among dietary guilds. We tested three primary hypotheses regarding the factors influencing sugar metabolism in Neotropical bats: (I) Fasting glucose levels are primarily determined by phylogenetic relatedness and dietary specialization, rather than individual characteristics such as sex or body mass; (II) Sugar assimilation responses in bats differ according to sugar type (monosaccharide versus disaccharide), reflecting differences in digestive and metabolic processing pathways. Besides, these responses vary among species according to dietary preferences, between sexes, and across temporal scales (diel and seasonal cycles); and (III) syntopic congeneric species exploiting similar food resources may differ in glucose assimilation and regulation, potentially reflecting physiological differentiation associated with resource partitioning to minimize competition for resources. We predicted that interspecific differences in fasting glucose levels would primarily be associated with diet and phylogenetic relatedness. Besides, we expected sugar assimilation patterns to vary by sugar type, dietary preferences, sex, time of day, and season [24], with differences between rainy and dry periods. Finally, we expected that syntopic species would differ in sugar assimilation and regulation, and that physiological differentiation would be greater among morphologically similar syntopic congeneric species that exploit similar food resources.

## Results

**Fig 1:**
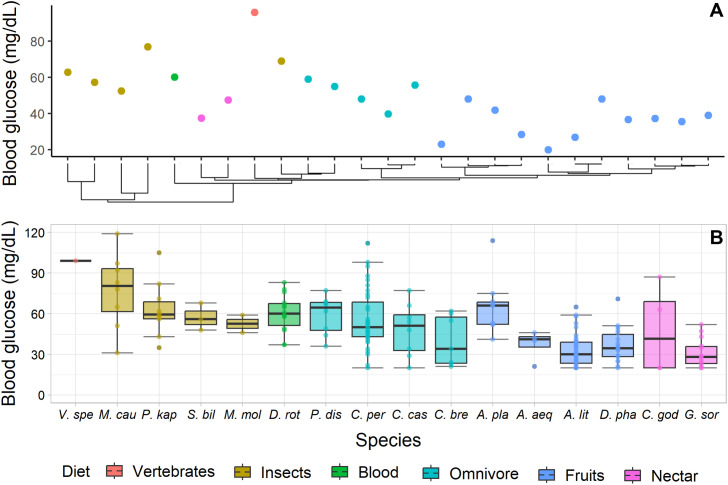
Fasting glucose levels in Neotropical bats. A) Average fasting glucose levels related to phylogeny, represented by 25 species and B) glucose levels associated with diet. In B the species are arranged by diet. Evolutionary relationships and diet influence fasting glucose levels across species, not sex or weight. See S1 Fig for species ID and sample sizes and S2 Fig for sex and weight relation to fasting glucose levels.

To test whether fasting glucose levels are primarily determined by phylogenetic relatedness and dietary specialization rather than individual characteristics, we investigated the effects of food preferences, sex, and body mass on fasting glucose levels across species (Fig 1). Our Bayesian multi-level phylogenetic model revealed that diet significantly affects fasting glucose levels in bats, as indicated by 95% credible interval estimates for the model’s parameters (S4 Fig). Specifically, bats that feed on nectar and fruits have lower fasting levels than those that consume animals. Furthermore, we detected a strong phylogenetic signal in fasting glucose levels (λ = 0.779; p = 0.021), indicating that closely related species exhibit more similar fasting glucose levels than species selected randomly. This phylogenetic signal suggests that fasting glucose regulation has evolved along lineages and is constrained by evolutionary history. In contrast, neither sex nor body mass showed significant effects on fasting glucose levels, suggesting that, despite high individual-level variation, fasting glucose varies with diet and phylogeny. However, because dietary specialization is often phylogenetically conserved in bats, the effects of diet and phylogenetic relatedness on glucose metabolism may be strongly correlated and difficult to disentangle completely.

**Fig 2:**
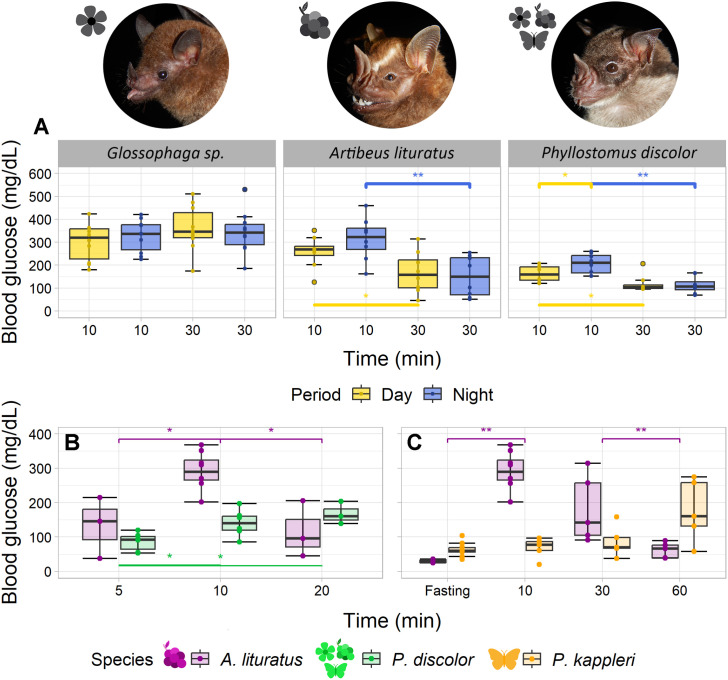
Sucrose assimilation in Neotropical bats. A) Comparison of sucrose assimilation between day and night, B-C) Analysis at various time points (0, 5, 10, 20, 30, and 60 minutes after sugar ingestion) for bats with different diets. The species studied include the nectar bat *Glossophaga sp.* (n = 10), the fruit bat *Artibeus lituratus* (A, n = 8; B, n = 8; C, n = 8), the omnivore *Phyllostomus discolor* (A, n = 8; B, n = 6), and the insectivore *Peropteryx kappleri* (n = 10).

We investigated whether sugar assimilation capacity differs between sexes and varies with temporal factors (diel and seasonal cycles), sugar type (monosaccharide versus disaccharide), and food preferences. Here, we measure sugar assimilation as the increase and decrease in blood glucose following a sugar meal, reflecting sugar absorption and the subsequent clearance of glucose from the blood. First, to determine the optimal time of day for measuring sugar assimilation, we measured glucose levels at 10 and 30 minutes after sucrose ingestion, with measurements conducted in the morning and after sunset (Fig 2A). We used sucrose because the organism must break it down into glucose and fructose before absorption [15], which results in lower glucose absorption than with pure glucose ingestion [9], but it allows us to standardize the process by accounting for the complexity of sugar digestion, including enzymatic activity [10,25]. After 30 minutes of sucrose ingestion, we observed that glucose levels in the three species were similar during the day and the night. However, 10 min after sugar ingestion, the glucose levels were higher at night in the omnivore *P. discolor* than during the day, and they tended to be higher in the frugivorous bat *A. lituratus*. In contrast, the species of *Glossophaga*, which have a dietary preference for nectar, showed similar glucose levels between 10 and 30 minutes, whether it was day or night, while the frugivore and omnivorous bats exhibited the highest glucose levels 10 minutes after sugar ingestion. Thus, our findings indicate that bats such as *Artibeus* and *Phyllostomus*, with frugivorous and omnivorous food preferences, exhibit a greater capacity to reduce blood glucose levels at night, with higher blood glucose levels at 10 minutes than at 30 minutes, regardless of the time of day.

We also evaluated sexual differences in *A. lituratus* and seasonal variations in sugar assimilation in *C. perspicillata*. In *A. lituratus*, females and males showed similar levels of assimilation for all three sugars (Fig 4B). On the other hand, *C. perspicillata* exhibited seasonal differences in assimilation only for sucrose, showing higher blood glucose levels during the rainy season than in the dry season (Fig 4C). Despite these fluctuations, the assimilation pattern remained consistent across both seasons, with an absorption peak at 10 minutes, and a decline at 30 and 60 minutes post-sucrose ingestion. For trehalose, *C. perspicillata* exhibited a seasonal pattern opposite to that of sucrose, with higher blood glucose levels during the dry season than during the wet season. Regarding glucose, during the dry season, the bats showed an absorption peak 10 minutes after ingestion, followed by a decrease in blood glucose levels. However, the average blood glucose levels were similar during the rainy season at 10 and 30 minutes after glucose consumption (Fig 4C).

**Fig 3:**
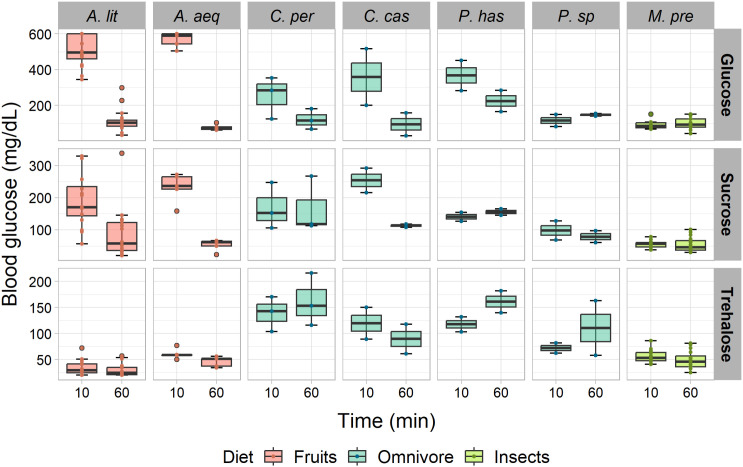
Intraindividual variation in the assimilation of three sugars by bats with different diets. The figure illustrates the assimilation of glucose, sucrose (found in fruits and nectar), and trehalose (found in insects’ hemolymph) by Neotropical bats with varying diets. The bats represented are: *A. lit: Artibeus lituratus* (n = 16), which shows differences in glucose (p-value 0.00043) and sucrose (p-value 0.00613) assimilation; *A. aeq: Artibeus aequatorialis* (n = 5)*, C. per: Carollia perspicillata* (n = 3)*, C. cas: Carollia castanea* (n = 2)*, P. has: Phyllostomus hastatus* (n = 2)*, P.* sp*.: Phyllostomus* sp. (n = 2)*, and M. pre: Molossus pretiosus* (n = 9), which exhibits differences in trehalose assimilation (p-value 0.02289).

To test whether sugar assimilation capacity varies with dietary guild, we examined differences in the absorption of three sugar types that predominate in the food of bats with distinct diets. Specifically, we tested the assimilation of glucose (a monosaccharide), sucrose (a disaccharide found in nectar and fruits), and trehalose (a disaccharide present in insects’ hemolymph). To determine optimal times for assessing sugar assimilation, we measured post-ingestion glucose levels at 5, 10, and 20 minutes after sucrose ingestion, revealing that 10 minutes was most informative by showing an assimilation peak in the frugivorous bat *A. lituratus* (Fig 2B). Extended measurements at 10, 30, and 60 minutes (Fig 2C) confirmed the 10-minute peak in frugivorous bats, which returned to baseline after 60 minutes. Conversely, the insectivorous bat *P. kappleri* exhibited delayed absorption, peaking at 60 minutes, reflecting dietary-specific metabolic adaptations. Based on previous results, we measured blood glucose levels at key time points (10 and 60 min) after feeding the same individual bats with each type of sugar (Fig 3). Each sugar was measured after a 5-hour interval, allowing blood glucose levels to return to fasting levels, even in insectivorous bats after sucrose consumption (S3 Fig). We found no intraspecific variation in the assimilation patterns for the three sugars; as expected, bats exhibited notably higher blood glucose levels when fed glucose than the disaccharides.

Frugivorous bats reached assimilation peaks more rapidly and attained exceptionally high blood glucose levels for both glucose and sucrose compared to omnivores and insectivores; differences were significant across all pairwise comparisons except the omnivore *Carollia castanea* (Fig 3). Omnivore bats showed variable sugar assimilation patterns. *Carollia* species and *Phyllostomus hastatus* exhibited faster glucose assimilation than *Phyllostomus* sp*.,* while C. *castanea* assimilated sucrose and trehalose faster than *C. perspicillata*, *P. hastatus*, and *Phyllostomus* sp. Blood glucose levels typically decline by 60 minutes post-glucose ingestion (except *Phyllostomus* sp.), whereas trehalose produced elevated levels at 60 minutes relative to other dietary groups. Sucrose assimilation displayed high intra- and interspecific variability (Fig 3). In *C. perspicillata*, two individuals showed elevated glucose levels at 60 minutes, while one showed reduced levels. In *P. hastatus*, responses were similarly divided. In contrast, all *C. castanea* and *Phyllostomus* sp individuals showed reduced glucose levels 60 minutes after sucrose consumption. Following trehalose ingestion, omnivorous bats exhibited higher glucose levels than frugivore bats and the insectivore *Molossus pretiosus* (Fig 3), whereas no differences were observed between the frugivore *A. aequatorialis* and the insectivore *M. pretiosus* species. Insectivorous bats exhibited lower blood glucose levels after consuming glucose and sucrose compared to other dietary guilds, with *Molossus* individuals showing limited capacity to assimilate all three sugar types in the 60 min window. In summary, frugivorous bats demonstrated enhanced glucose and sucrose assimilation capacity, while omnivores showed the highest trehalose assimilation and significant variation in glucose and sucrose assimilation.

**Fig 4:**
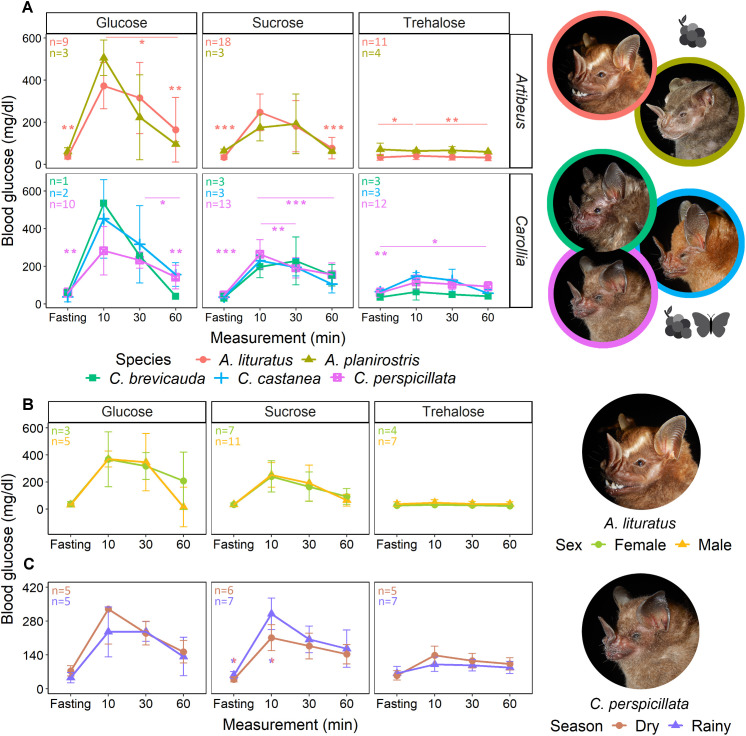
Glucose, sucrose, and trehalose tolerance tests for syntopic species, comparing sexes and seasonal assimilation. A) congeneric syntopic species of the frugivorous bat *Artibeus*; the bigger *A. lituratus* (forearm: 68-77 mm) and *A. planirostris* (forearm: 62-70 mm), and the frugivorous/omnivorous *Carollia*; the smaller *C. castanea* (forearm: 34-37 mm), and the bigger *C. brevicauda* (forearm: 38-41 mm) and *C. perspicillata* (forearm: 39-44 mm), B) *Artibeus lituratus* females and males, C). *Carollia perspicillata* during the dry and rainy seasons.

Finally, we tested whether syntopic congeneric species have metabolic differences associated with ecological niche differentiation and resource partitioning (Fig 4A). For the frugivorous species of the genus *Artibeus*, blood glucose levels remained nearly constant after trehalose ingestion. However, *A. planirostri*s showed higher glucose levels than *A. lituratus* following glucose ingestion, while only *A. lituratus* exhibited a slight increase in blood glucose levels after trehalose ingestion. For the omnivorous species of *Carollia* genus, *C. brevicauda* showed the lowest trehalose assimilation, *C. perspicillata* exhibited the lowest glucose levels after glucose ingestion, while *C. castanea* showed the highest levels following trehalose consumption.

## Discussion

Fasting glucose levels varied significantly among dietary guilds (lower in plant- than in animal consumers), showed a strong phylogenetic signal, and were independent of sex and body mass. After feeding with glucose and sucrose, frugivores have rapid assimilation (10-minute peaks). On the other hand, omnivores showed variable but superior trehalose processing, whereas the insectivore *M. pretiosus* exhibited delayed, limited absorption across all sugars. As expected, these patterns align with differences in sugar composition between diets—sucrose predominates in fruits/nectar, trehalose in insects—indicating adaptive metabolic specialization as previously shown [9]. Additionally, we observed intraindividual variation in metabolic responses to different sugar types. Temporal variation in sugar processing occurred primarily at diel scales, with enhanced nocturnal absorption in frugivores and omnivores, while seasonal effects were sugar-specific in the omnivore *C. perspicillata*. Syntopic congeners showed subtle metabolic differentiation, suggesting physiological niche partitioning, whereas sex differences were absent in the species we tested: frugivore *A. lituratus*. These findings reveal that bat sugar metabolism reflects evolutionary adaptation to dietary niches, constrained by phylogeny and modulated by circadian rhythms and probably seasonality, with minimal influence from intrinsic factors.

### Fasting glucose levels

Neotropical bats exhibited fasting responses associated with diet, which is consistent with our predictions [26]. Similar patterns have been documented in Old World species [17,27], where bats consuming protein-rich insects or carbohydrate-rich fruits maintain constant glucose levels during fasting through metabolic reserves, whereas nectar-feeding bats that rely on continuous sugar intake showed an inability to regulate glucose after short-term fasting. Vampire bats (*Desmodus rotundus*) are particularly vulnerable to fasting stress [28], showing higher fasting blood glucose levels than frugivorous and nectarivorous species after fasting periods, potentially reflecting the use of glycogen. These bats maintain glucose levels compatible with most mammals (over 60 mg/dL) after a 12-hour fasting period [29]. However, after 24 hours of fasting, their glucose levels can drop significantly to around 30 mg/dL, indicating a potential loss of their ability to tolerate long-term fasting [28]. Nectarivorous bats also exhibit susceptibility to fasting [30], as evidenced by their low glucose levels after short-term fasting. This vulnerability reflects limited energetic reserves [30] since the energy extracted from sugar is primarily allocated to meet the high metabolic demands of flight [31] rather than stored for future use. Interestingly, frugivorous bats exhibited fasting glucose levels comparable to those of nectarivorous species after the same fasting period (~ 50 mg/dL or lower). These results contrast with captive *Artibeus* that maintained stable blood glucose levels (~ 100 mg/dL) after a 24-hour fasting period, declining to ~ 45 mg/dL only after a 72-hour and a six-day fasting period [32]. This discrepancy likely reflects methodological differences since, in the mentioned study, captive bats received ultra-rich sugar diets (bananas, watermelon, papayas, etc.) for one week before fasting trials, accumulating fat reserves, and experienced reduced metabolic demands due to a more sedentary lifestyle [32] compared to the recently captured free-flying bats tested in our study. Finally, insectivorous and carnivorous bats exhibited higher glucose levels after 12 hours of fasting, consistent with patterns observed in vertebrates consuming high-protein diets [33,34] and distinct from those of other dietary guilds. Therefore, these patterns suggest that dietary specialization drives divergent fasting strategies in Neotropical bats, with animalivorous protein consumers showing the greatest tolerance, frugivores intermediate capacity, and nectar specialists’ acute vulnerability, reflecting evolutionary trade-offs between metabolic efficiency for preferred resources and physiological buffering during scarcity.

In non-reproductive individuals, sex did not significantly affect fasting glucose levels, consistent with previous studies [28,35]. However, sex-specific differences may emerge during longer fasting periods: male *Molossus molossus* maintained stable glucose levels, whereas females showed declining levels after 48 hours of food deprivation [36]. This result suggests that sexual differences, with larger males of *M. molossus* exhibiting superior glucose regulation compared to smaller females, may be indicate metabolic differentiation and resource partitioning between sexes. This hypothesis warrants investigation in other sexually dimorphic bat species with documented dietary segregation, which could reveal whether physiological niche differentiation reinforces sexual divergence in body size and ecology. Body mass showed no clear association with fasting glucose levels across all species (Fig 1C). However, previous research reported that smaller frugivorous bats have higher glucose levels than larger species [17], a pattern we observed within frugivore-omnivore genera (such as *Artibeus and Carollia*, S2 Fig) but not across the Stenodermatinae subfamily (Fig 1A). In humans, diet, activity, and body mass interactively influence fasting glucose levels [37]; therefore, the inconsistent association between body size and glucose regulation may reflect complex interactions among these factors rather than the isolated influence of body mass alone. Besides, the effect of body size on fasting glucose could be further explored by adding bats with higher body masses to the datasets.

### Variation in sugar assimilation

Omnivorous and frugivorous bats exhibited the highest sugar assimilation capacity during the night, whereas nectarivorous species showed similar assimilation rates between day and night. The rapid nocturnal glucose level elevation observed in *Phyllostomus* and *Artibeus* is consistent with their activity patterns [37] and reflects efficient carbohydrate assimilation for flying and foraging during nighttime periods [38]. In contrast, nectarivorous *Glossophaga* species maintain minimal energy reserves [30] and rely primarily on exogenous glucose intake from their diet, using circulating blood glucose as their immediate energy source to sustain activity regardless of the time of day [31,39]. Therefore, they may rely on exercise to trigger sugar assimilation during the night [16]; thus, we probably did not find differences here because we maintained bats at rest during our sampling.

Sucrose assimilation capacity differed significantly between bats with high (frugivorous) and low-sugar diets (insectivorous) at 10 and 60 minutes post-ingestion (Fig 2C), reflecting diet-specific physiological and anatomical adaptations. Frugivorous species possess: (i) elongated duodenum providing greater surface area for paracellular glucose diffusion, (ii) intestinal glucose transporters enhancing transcellular glucose absorption, and (iii) elevated sucrase expression enabling rapid disaccharide hydrolysis [9,10,14].

The temporal dynamics of sugar assimilation in the frugivorous bat *A. lituratus* revealed no detectable glucose absorption peak within 5 minutes following sucrose ingestion, suggesting that the initial digestive and absorption process required for disaccharide hydrolysis and glucose assimilation may take at least 10 minutes [15]. For sucrose, peak absorption occurred at 10 minutes in *A. lituratus*, followed by declining levels at 20 minutes as glucose was transported from the blood to tissues [40], and returned to fasting levels by 60 minutes. Notably, insectivorous bats showed peak glucose levels at 60 minutes, when frugivores had already returned to baseline. These contrasting temporal assimilation profiles, with 10 and 60 minutes emerging as critical assessment points, have been documented across several bat species and sugar types [9].

To compare the assimilation of the three sugars (sucrose, glucose, and trehalose) among bats with different diets, we fed each individual with all three sugars separately. Results aligned with previous reports of assimilation capacity and enzymatic activity [9,10], though omnivorous bats showed greater variability than previously documented [9]. Frugivorous bats demonstrated high glucose and sucrose assimilation but slow trehalose processing, consistent with their sugary-rich fruit-based diets. Limited trehalose assimilation was expected, as the *treh* gene encoding trehalase is pseudogenized in non-insectivorous bats [9,41], and trehalase activity decreased during evolutionary transition from insectivory to other diets [10]. Conversely, the insectivorous *Molossus pretiosus* exhibited minimal sugar assimilation across all three substrates, with no significant changes in blood glucose following ingestion. *Molossus* species feed primarily on coleopterans [41,42], which are rich in proteins and lipids rather than carbohydrates [43,44], suggesting that their digestive physiology is specialized for protein and lipid metabolism rather than sugar assimilation [10]. In contrast, omnivorous bats exhibited substantial intra- and interspecific variation in sugar assimilation. This heterogeneity likely reflects: i) variations among species in the degree of omnivory as a result of intermediate physiological and metabolic adaptations between faunivorous and herbivorous dietary extremes [45], and ii) high physiological plasticity in omnivorous species, which enables flexible metabolic responses to variation in diet composition and resource availability, even at the individual level [45–47].

*Artibeus* and *Carollia* showed similar glucose and sucrose assimilation patterns (Fig 4A), despite distinct fruit preferences: species of *Artibeus* primarily consume figs and *Cecropia*, while *Carollia* preferentially feed on fruits of *Piper* plants [48]. Although these fruits differ in nutritional composition [49,50], all are carbohydrate-rich, producing a *Fast* assimilation pattern [9] with high glucose digestive efficiency [50] and sugar-rich diet adaptations. Trehalose assimilation, however, revealed notable metabolic differences in the assimilation between genera. Species of *Carollia* exhibited higher trehalose assimilation than species of *Artibeus*, consistent with previous findings on these genera [9]. This ability to digest trehalose, a sugar commonly found in insects, is likely related to the significant consumption of insects in the diet of *Carollia* species [51]. While *Carollia* species can assimilate all three sugar types, *Artibeus* shows specialization primarily for fruit-derived sugars, suggesting *Carollia* maintains broader metabolic flexibility for the assimilation of diverse carbohydrates.

Male and non-reproductive female *A. lituratus* individuals showed similar patterns of sugar assimilation (Fig 4B). However, reproductive status may reveal sex-specific metabolic differences, as reproduction imposes substantial energetic demands on individuals of this genus [52], potentially creating temporally divergent metabolic requirements between males and females that warrant further investigation. Besides, future studies should examine whether sex-specific sugar assimilation emerges in species exhibiting sexual size dimorphism or sex-based dietary partitioning, which does not apply to species of *Artibeus* [53].

The omnivorous species *C. perspicillata* is a primary consumer of *Piper* fruits that showed higher sucrose assimilation during the rainy season than during the dry season (Fig 4C), evidenced by a higher glucose peak at 10 minutes post-ingestion, indicating enhanced sucrose hydrolysis and glucose absorption through the small intestine [15]. This seasonal metabolic change aligns with plant phenology in dry tropical forests, with most animal-dispersed plants fruiting during the rainy season [54], including synchronized fruiting peaks in *Piper* species [55]. Thus, elevated sucrose processing capacity in *Carollia* coincides temporally with peak availability of their primary food resource, suggesting metabolic upregulation in response to seasonal resource abundance. Conversely, *C. perspicillata* exhibited a slight increase in trehalose assimilation during the dry season, potentially reflecting a metabolic adjustment associated with a dietary shift toward greater consumption of nocturnal insects when fruit availability declines in the forest understory [56,57]. Thus, this pattern suggests a potential seasonal dietary flexibility [58–60], with *C. perspicillata* adjusting foraging effort to exploit the most available resources [61]. Given documented seasonal shifts in nectar-feeding bat behavior and dietary flexibility in related insectivorous species in seasonal ecosystems [62], further research should examine how bat carbohydrate metabolism associates with seasonal resource availability [63,64]. Such studies will enhance our understanding of physiological plasticity and species vulnerability to habitat degradation and climate change.

### Metabolic differentiation between syntopic congeneric species

To test whether syntopic congeneric species exhibit metabolic differentiation, potentially associated with food resource partitioning, we compared sugar assimilation among three *Carollia* species: *C. perspicillata*, *C. brevicauda*, and *C. castanea*. Despite morphological and ecological similarity, these three species coexist through niche partitioning, including varying degrees of insectivory versus frugivory [51]. Our results reveal metabolic differentiation, suggesting resource partitioning. Among the species with similar body sizes, *C. brevicauda* showed high glucose but low trehalose assimilation, suggesting specialization on fruit-derived sugars. Conversely, *C. perspicillata* exhibited lower glucose but higher trehalose assimilation than *C. brevicauda*, suggesting greater reliance on insect-derived sugars. This physiological divergence requires further confirmation by increasing the sample size. However, it is potentially associated with food resource segregation, which reduces competition by reinforcing dietary preferences despite similar body size and external morphology, with metabolic capacity possibly tracking resource-use patterns. *C. castanea*, the smallest congener, showed high assimilation of all three sugars, suggesting broader metabolic flexibility. This may reflect a different niche partitioning mechanism: a small body size and morphological differentiation in cranial and dental traits enable access to distinct food items, reducing reliance on metabolic specialization for competitive exclusion. These patterns are consistent with the possibility that metabolic differentiation facilitates coexistence among morphologically similar congeners even in habitats with limited food resources, while morphologically divergent species may rely more strongly on metabolic flexibility. If confirmed across additional species and systems, this would suggest that physiological specialization in food processing emerges as an adaptive response when other mechanisms for niche partitioning are constrained.

## Materials and methods

### Capturing bats

We caught bats using mist nets set up in dry and humid forest understories in Colombia’s Valle del Cauca and Chocó departments between 2019 and 2020. The nets were active from 18:30–21:00 h and were checked every 30 minutes. We ensured that all captured bats were fed before a 10–12-hour fast post-capture. For taxonomic identification, we used a taxonomic key for South American bats [65]. We recorded the sex of the individuals and assessed the biological age by using transillumination of their metacarpal-phalangeal joints [66]. In addition, we evaluated the reproductive status of the bats, including only non-reproductive adults (applicable to females) in our study to minimize physiological variation that can occur due to growth, or increased stress during pregnancy, and lactation [7,20,67].

### Dietary classification

We classified the species based on their dietary preferences obtained by previous research [68], examinations of teeth and cranial morphology [68–71], tongue and gut anatomy [72]. These traits relate to how species acquire, process, and assimilate food. We considered six species as primarily insect consumers (*Peropteryx kappleri*, *Saccopteryx bilineata*, *Molossus molossus*, *Molossus pretiosus*, *Myotis*, *Garnerycteris crenulatum*), one species as a blood feeder (*Desmodus rotundus*), one as a carnivorous (*Vampyrum spectrum*), two as nectar consumers (*Glossophaga soricina*, *Choeroniscus godmani*), eleven as preferring fruits (*Sturnira ludovici*, *S. luisi*, *S. parvidens*, *S. giannae*, *Platyrrhinus helleri*, *Uroderma bakeri*, *U. convexum*, *Dermanura* sp., *Artibeus aequatorialis*, *A. planirostris*, *A. lituratus*), and six as omnivores (*Phyllostomus discolor*, *P. hastatus*, *Carollia castanea*, *C. brevicauda*, *C. perspicillata*), consuming primarily at least two different food types and showing trehalose assimilation [9].

### Blood glucose measurements

We assessed sugar assimilation by measuring the increase in blood glucose concentration after feeding each individual a specific type of sugar, in accordance with the objectives of our tests. We measured blood glucose levels using a single drop of blood collected from the forearm of each individual with a lancet and a glucometer (Glucoquick G30a, Diabetrics®). The individuals were fed with a 20% sugar solution (glucose, sucrose, or trehalose) after a fasting period of 10–12 hours to record their baseline glucose levels. Following this, we measured blood glucose levels at 5, 10, 20, 30, and 60 minutes after ingesting a single 20% glucose solution. The amount of sugar provided to bats (5.4 g/kg) was calculated based on their body weight according to [73]. We tested the assimilation of glucose and sucrose—a monosaccharide and a disaccharide commonly found in fruits and nectar [11]—and trehalose, a disaccharide found in insects’ hemolymph [12], while bats were at rest in soft cloth bags. This methodology was followed both during the dry and the rainy seasons for several individuals of *Carollia perspicillata* since dietary shifts related to seasonality [59] could impact sugar assimilation.

To standardize the optimal time for measuring glucose levels, we offered sucrose to bats with different diets and compared their blood glucose levels 10 and 30 minutes after ingestion, both day and night. We observed a similar assimilation pattern between day and night, so the remaining assimilation tests were conducted from sunrise to sunset. On the other hand, to identify the most informative time for assessing sugar assimilation, we measured glucose levels at 5, 10, 20, 30, and 60 minutes after the bats consumed a disaccharide (sucrose), which requires an additional enzymatic step for assimilation compared to monosaccharides [15]. Our findings indicated that measuring glucose levels 10 minutes after feeding bats with a sugar-rich diet (like frugivores) provided helpful information, reflecting an absorption peak. In contrast, we observed an absorption peak for insectivorous bats 60 minutes after sugar ingestion.

To ensure consistency, we first measured the assimilation of the three sugars (glucose, sucrose, and trehalose) in each individual at two-time points: 10 and 60 minutes. This approach allowed us to control for intraspecific variation. Each sugar was offered separately, with a 5-hour interval between offerings, sufficient for the blood glucose levels of both frugivorous and insectivorous bats to return to baseline (S3 Fig). Additionally, we measured the assimilation of just one sugar (glucose, sucrose, or trehalose) per individual of the most frequently captured species at various time points throughout the hour (0, 10, 30, and 60 minutes). This method enabled us to compare glucose levels while minimizing the risk of excessive blood sampling from any bat.

The data included in the analysis and figures of the paper can be found at https://github.com/andreaber1/sugar_metabolism_bats.

### Statistical analysis

To assess the effects of sex, weight, and diet on fasting glucose levels in bats, we performed a Bayesian multi-level phylogenetic model using the brms R package [74]. This model used blood glucose concentrations measured after fasting and incorporated a phylogeny that included multiple families and 16 species [75]. To account for both multiple individuals within species and shared evolutionary history, we included a species-level random intercept with a phylogenetic covariance structure based on the species tree, assuming Brownian motion evolution. This approach models observations as nested within species and incorporates phylogenetically structured covariance among species, thereby controlling for non-independence arising from both within-species pseudoreplication and phylogenetic relatedness. Additionally, we evaluated the phylogenetic signal of fasting glucose levels with Pagel’s lambda [76]. Furthermore, to individually assess differences in sugar assimilation among frugivore bats based on intra- or inter-genera comparisons, time of day (day/night), sex, and season, we employed the Wilcoxon test. This test allowed us to compare blood glucose levels across different time points and treatment conditions. All the statistical analyses were performed using R 4.3.1 [77].

### Ethical statement

This research was authorized by the Colombian National Environmental Authorities: Autoridad Nacional de Licencias Ambientales and Ministerio del Ambiente y Desarrollo Sostenible (Permit 1070). Anesthesia, analgesia, or euthanasia were not required or applied during the study.

## Supporting information

S1 Fig Fasting blood glucose levels and phylogenetic relations with species names. Average fasting blood glucose levels are related with the phylogeny [75] showing the species identification. From left to right, *Peropteryx kappleri* (n = 10)*, Saccopteryx bilinieata* (n = 3)*, Molossus molossus* (n = 2)*, Myotis sp.* (n = 8), *Desmodus rotundus* (n = 16)*, Glossophaga soricina* (n = 20)*, Choeroniscus godmani* (n = 4)*, Vampyrum spectrum* (n = 1)*, Gardnerycteris crenulata* (n = 1)*, Phyllostomus discolor* (n = 8)*, P. hastatus* (n = 1)*, Carollia castanea* (n = 8)*, C. brevicauda* (n = 7)*, C. perspicillata* (n = 35)*, Sturnira ludovici* (n = 1)*, S. luisi* (n = 1)*, S. parvidens* (n = 1)*, S. giannae* (n = 2)*, Platyrrhinus helleri* (n = 1)*, Uroderma bakeri* (n = 2)*, U. convexum* (n = 1)*, Dermanura sp.* (n = 18)*, Artibeus aequatorialis* (n = 4)*, A. planirostris* (n = 10)*, and A. lituratus* (n = 55).
(TIFF)

S2 Fig Fasting blood glucose levels related to weight and sex. **A). in *Carollia perspicillata* (n = 35) and *Artibeus lituratus* (n = 42), and B). in 16 species.** On average, *A. lituratus* shows lower levels than *C. perspicillata,* but there is no significant difference between the sexes within species.
(TIFF)

S3 Fig Timepoints between blood glucose measurements. Measurements to ensure fasting glucose levels between sugar assays. We found that 4–5 h were enough to ensure decreased blood glucose levels reaching fasting glucose levels after disaccharide (sucrose) consumption for frugivorous, omnivorous, and insectivorous bats. There were no significant differences between timepoints within the same species. *A. lituratus* (n = 12, 5 per timepoint respectively), *P. discolor* (n = 4), *P. kappleri* (n = 10, 2, 5 per timepoint respectively).
(TIFF)

S4 Fig Bayesian multi-level phylogenetic model for fasting glucose levels. Bayesian multi-level phylogenetic model 95% confidence intervals testing fasting glucose levels for bats with A). variable composition of sugar in their diets (phytophagous with high sugar content in their diet n = 116, animalivores with low sugar content in their diet n = 35, and omnivores with varied content of sugar in their diet n = 84), B). different sexes, males n = 131, females n = 57, and C). distinct body weights n = 188. There is a tendency for lower fasting blood glucose levels in bats with sugar-rich diets (nectarivores and frugivores) compared to bats with rich-protein diets (insectivores and omnivores).
(TIFF)
